# Machine Learning Techniques Used for the Identification of Sociodemographic Factors Associated With Cancer: Systematic Literature Review

**DOI:** 10.2196/79187

**Published:** 2026-01-28

**Authors:** Liz González-Infante, Gaston Marquez, Solange Parra-Soto, Mónica Cardona-Valencia, Carla Taramasco

**Affiliations:** 1 Facultad de Ciencias Empresariales, Universidad del Bío-Bío, Andrés Bello 720, Chillán, Chile, 56 422463324; 2 Centro para la Prevención y el Control del Cáncer, Santiago, Chile; 3 Departamento de Ciencias de la Computación y Tecnologías de la Información, Facultad de Ciencias Empresariales, Universidad del Bío-Bío, Chillan, Chile; 4 Departamento de Nutrición y Salud Pública, Facultad Ciencias de la Salud y de los Alimentos, Universidad del Bío-Bío, Chillán, Chile; 5 Departamento Ciencias de la Rehabilitación en Salud, Facultad de Ciencias de la Salud y de los Alimentos, Universidad del Bío-Bío, Chillán, Chile; 6 ITISB, Facultad de Ingeniería, Universidad Andrés Bello, Viña del Mar, Chile

**Keywords:** cancer, health disparities, machine learning, predictive models, social determinants of health, sociodemographic factors, systematic review

## Abstract

**Background:**

Cancer remains one of the foremost global causes of mortality, with nearly 10 million deaths recorded by 2020. As incidence rates rise, there is a growing interest in leveraging machine learning (ML) to enhance prediction, diagnosis, and treatment strategies. Despite these advancements, insufficient attention has been directed toward the integration of sociodemographic variables, which are crucial determinants of health equity, into ML models in oncology.

**Objective:**

This review aims to investigate how ML techniques have been used to identify patterns of predictive association between sociodemographic factors and cancer-related outcomes. Specifically, it seeks to map current research endeavors by detailing the types of algorithms used, the sociodemographic variables examined, and the validation methodologies used.

**Methods:**

We conducted a systematic literature review in accordance with the PRISMA (Preferred Reporting Items for Systematic Reviews and Meta-Analyses) guidelines. Searches were executed across 6 databases, focusing on the primary studies using ML to investigate the association between sociodemographic characteristics and cancer-related outcomes. The search strategy was informed by the PICO (population, intervention, comparison, and outcome) framework, and a set of predefined inclusion criteria was used to screen the studies. The methodological quality of each included paper was assessed.

**Results:**

Out of the 328 records examined, 19 satisfied the inclusion criteria. The majority of studies used supervised ML techniques, with random forest and extreme gradient boosting being the most commonly used. Frequently analyzed variables include age, male or female or intersex, education level, income, and geographic location. Cross-validation is the predominant method for evaluating model performance. Nevertheless, the integration of clinical and sociodemographic data is limited, and efforts toward external validation are infrequent.

**Conclusions:**

ML holds significant potential for discerning patterns associated with the social determinants of cancer. Nevertheless, research in this domain remains fragmented and inconsistent. Future investigations should prioritize the integration of contextual factors, enhance model transparency, and bolster external validation. These measures are crucial for the development of more equitable, generalizable, and actionable ML applications in cancer care.

## Introduction

The use of machine learning (ML) in oncology has advanced significantly over the past decade, offering new opportunities for early detection, survival prediction, and treatment personalization. Models based on techniques such as random forests (RFs), extreme gradient boosting (XGBoost), and deep neural networks have demonstrated remarkable performance across different types of cancer, fueling enthusiasm for what has been termed digital precision oncology [1]. However, most of these applications rely almost exclusively on clinical and biomedical data, limiting their ability to capture the broader social and structural factors that shape health outcomes [2]. This gap raises important concerns, as it may compromise both the external validity and the equity of ML models. In this review, we consistently use the term sociodemographic factors to refer to variables such as age, male or female or intersex, educational attainment, income, ethnicity, rurality, and access to health care. These factors conceptually overlap with the broader category of social determinants of health (SDoH), but our focus is on those variables that are typically available in clinical and research datasets and are explicitly integrated into ML models. By doing so, we ensure clarity and terminological consistency throughout the paper.

Our review focuses on the most common sociodemographic variables in clinical and research datasets, such as age, male or female or intersex, education, income, and others, reflecting the current landscape of published ML studies rather than a deliberate theoretical choice. We recognize that these indicators only capture part of the social gradient influencing cancer outcomes. Therefore, we highlight the importance of future research integrating contextual and multilevel determinants, such as neighborhood characteristics, health care infrastructure, environmental exposures, and political factors, to promote an equity-centered approach to ML applications in oncology.

In parallel, the rise of explainable artificial intelligence (AI) has highlighted the importance of transparency and interpretability in clinical settings. Tools such as Shapley Additive Explanations and local interpretable model-agnostic explanations allow health care professionals to better understand ML models by identifying which variables are most relevant in predictions and how they interact with both clinical and sociodemographic factors [3]. These advances not only strengthen trust in ML-based systems but also enhance their potential for integration into clinical practice and public health policy [4]. The convergence of explainable AI and SDoH emerges as a promising pathway toward developing fairer and more actionable models.

Nevertheless, our review of the literature reveals that although research and reviews on ML in oncology are rapidly expanding, most have concentrated on methodological, genomic, or clinical aspects without adequately addressing sociodemographic factors. This omission limits the ability of the scientific community to develop robust guidelines for implementing models across diverse contexts and health systems. Against this backdrop, this study aimed to identify, characterize, and synthesize primary research that applied ML methods to analyze sociodemographic factors associated with cancer. The objective was to address both methodological and conceptual gaps while contributing to the development of fairer and more transparent models that can inform data-driven public health strategies. We present the results of a systematic literature review (SLR) examining how ML techniques have been used to identify and interpret sociodemographic factors in cancer-related studies. Of the 328 papers screened, 19 (5.8%) met the inclusion criteria. Rather than being a limitation, this number reflects the emerging nature of the field and highlights the value of conducting an early review to consolidate initial progress, make methodological and equity-related gaps more visible, and guide future research toward a stronger integration of sociodemographic factors in ML models applied to oncology.

## Methods

### Research Questions

Based on the main objective, we defined the following research questions:

1. What ML techniques have been applied in studies that analyze sociodemographic data of patients with cancer to identify factors associated with the disease?
2. What sociodemographic factors have been consistently identified as relevant to the diagnosis, progression, or treatment of cancer?

### Identification

The SLR was conducted in accordance with the PRISMA (Preferred Reporting Items for Systematic Reviews and Meta-Analyses) guidelines (Checklist 1), which provide a rigorous framework for ensuring transparency and reproducibility in evidence synthesis [5]. To guide the construction of the search strategy, we also adopted the PICO (population, intervention, comparison, and outcome) model, as recommended by Petersen et al [6]. This framework allowed us to clearly define the target population, specify the type of intervention (ie, application of ML techniques), and focus the outcome on the identification of relevant sociodemographic factors associated with cancer (Table 1).

**Table 1.** Keywords used in the PICO (population, intervention, comparison, and outcome) structure.

Component	Description	Keywords
Population	Studies analyzing data from patients with cancer that include sociodemographic variables. These may encompass age, male or female or intersex, socioeconomic status, education, and residence among others.	“Sociodemographic factors,” “social determinants,” “sociodemographic characteristics,” and “socio-demographic variables”
Intervention	Application of machine learning techniques to identify and analyze sociodemographic factors associated with cancer.	“Machine learning” and “artificial intelligence”
Comparison	No previous studies with similar scope and objectives were identified as suitable comparators. This review explores a novel approach.	Not applicable
Outcome	Identification of the most relevant sociodemographic variables associated with cancer outcomes, and assessment of the predictive performance of the applied machine learning models.	“Cancer,” “oncology,” variable importance, model accuracy, and AUC^a^

a AUC: area under the curve.

The search terms were combined using the Boolean operators AND and OR to ensure comprehensive retrieval of relevant literature. The final search string was as follows:

([“sociodemographic factors” OR “socio-demographic factors” OR “sociodemographic characteristics” OR “socio-demographic characteristics” OR “social determinants” OR “sociodemographic variables” OR “socio-demographic variables”) AND (“machine learning” OR “artificial intelligence”) AND (“cancer” OR “oncology”])

### Screening

We conducted a comprehensive literature search across 6 major databases: PubMed (n=76), ACM Digital Library (n=85), ScienceDirect (n=7), IEEE Xplore (n=1), Web of Science Core Collection (n=80), and Scopus (n=79). Searches covered the period from database inception to October 14, 2024. PubMed was selected as the primary source for biomedical and oncology research. ScienceDirect was included to capture papers published in Elsevier journals not indexed elsewhere. ACM Digital Library and IEEE Xplore were used to retrieve computer science and engineering studies, where ML methods are often first reported. Web of Science facilitated interdisciplinary retrieval and citation tracking, while Scopus provided broad multidisciplinary coverage.

All records were exported, merged, and deduplicated prior to screening. To maximize comprehensiveness and minimize selection bias, we also applied forward and backward citation chasing on included studies. Full electronic search strategies for each database are provided in Multimedia Appendix 1.

### Paper Selection

#### Eligibility Criteria

Primary studies were screened and selected based on predefined inclusion and exclusion criteria. The specific inclusion criteria applied are summarized in Textbox 1.

Textbox 1. **Inclusion and exclusion criteria.**

**Inclusion criteria**

- Type of study: primary studies presenting original data or analysis. Quantitative studies applying machine learning techniques to analyze sociodemographic factors related to cancer, including experimental, observational (cohort, case-control, and cross-sectional), or methodological designs.
- Study area: application of machine learning in health, focused on the analysis of sociodemographic factors (eg, age, male or female or intersex, ethnicity, socioeconomic status, and health care access) and their association with any type of cancer (eg, breast, lung, prostate, and gastrointestinal).
- Machine learning techniques: use of supervised algorithms (eg, neural networks, decision trees, support vector machines, and logistic regression), unsupervised (eg, clustering), or semisupervised algorithms. Reporting of performance metrics such as accuracy, sensitivity, specificity, and receiver operating characteristic area under the curve.
- Sociodemographic factors: explicit analysis of sociodemographic variables related to cancer risk, prevalence, or progression, including age, male or female or intersex, ethnicity, income, education, occupation, geographic location, health care access, and other socioeconomic determinants.
- Publication period: studies published from 2014 onward.
- Language: publications in English or Spanish.
- Accessibility: full-text access or access to essential data and results enabling methodological evaluation.

**Exclusion criteria**

- Type of study: systematic reviews, narrative reviews, meta-analyses, or secondary studies.
- Study area: studies not analyzing the association between sociodemographic factors and cancer. Studies focused on other diseases (eg, diabetes and cardiovascular diseases).
- Machine learning techniques: studies relying solely on traditional statistical methods and not reporting model validation metrics.
- Sociodemographic factors: studies applying machine learning without including sociodemographic variables (eg, focused only on genetic, molecular, or biological data).
- Publication period: Studies published before 2014.
- Language: publications in other languages without available translation.
- Accessibility: abstracts or conference proceedings without access to the full paper.

#### Quality Assessment

The purpose of the quality assessment was to evaluate the relevance of each selected paper. Although quality assessment did not influence the selection of primary studies [7], we included it primarily to reflect the validity of the selected studies. Based on the response to each research question, we scored each paper with 2, 1, or 0 points. We then selected those papers that exceeded the 50% threshold. The studies chosen through this assessment ensure that our conclusions, drawn from the extracted data, are supported by adequately resourced evidence (Multimedia Appendix 1).

#### Study Selection and Resolution of Discrepancies

Each paper was independently screened by 2 reviewers according to predefined inclusion and exclusion criteria. Any disagreements regarding eligibility were addressed during consensus meetings, where reviewers jointly discussed the rationale for inclusion or exclusion. When consensus could not be reached, a third author was consulted to make the final decision. This procedure ensured transparency, reproducibility, and rigor throughout the study selection process.

## Results

### Overview

**Figure 1.**
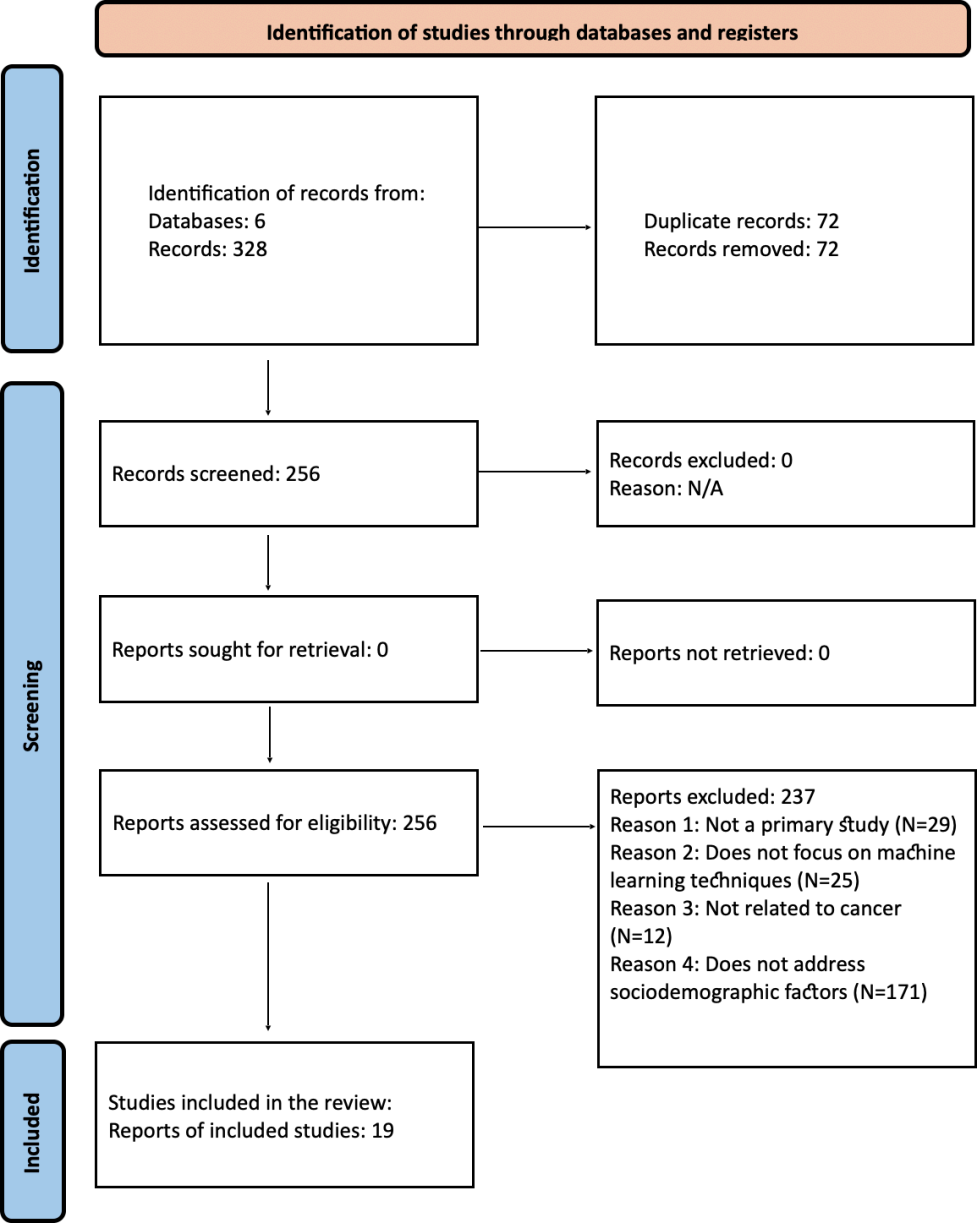
PRISMA (Preferred Reporting Items for Systematic Reviews and Meta-Analyses) flowchart of the selection of primary studies for the systematic literature review. N/A: not applicable.

The SLR was conducted in accordance with the PRISMA guidelines, which provide a rigorous framework for ensuring transparency and reproducibility in evidence synthesis (Figure 1). Following the PRISMA methodology, a total of 15 primary studies published in peer-reviewed journals were identified. An additional 4 papers were included through forward snowballing, yielding a final sample of 19 studies. Among these, 58% (11/19) were conducted in the United States. Iran contributed 21% (4/19), followed by India with 11% (2/19), and South Korea with 5% (1/19). One study (5%) represented a collaborative effort between institutions in China and the United States (Table 2). The publication dates of the included studies ranged from 2018 to 2024. No eligible primary studies were found in workshop proceedings or book chapters.

**Table 2.** Distribution of primary studies by country.

Country	Number of studies
United States	11
Iran	4
India	2
South Korea	1
China-US collaboration	1

### Machine Learning Algorithms and Validation Strategies Reported

Across the studies analyzed, consistent patterns emerged in both the selection of ML algorithms and the validation methods used (Table 3).

**Table 3.** Summary of the machine learning algorithms and validation strategies reported across the 19 primary studies. Most studies applied ensemble methods such as random forest (RF) or gradient boosting, frequently combined with cross-validation schemes.

Study ID	Algorithms used	Validation strategy	Reference
S1	Lasso^a^ LR^b^, RF, gradient boosting, DT^c^, SVM^d^	5-fold CV^e^, ROC-AUC^f^, accuracy, sensitivity, specificity	[8]
S2	XGBoost^g^, LightGBM^h^, CatBoost^i^, RF, AdaBoost, Lasso regression	10-fold CV	[9]
S3	DT, RF	10-fold CV	[10]
S4	RF, artificial neural networks, bootstrap aggregating CART^j^, XGBoost	10-fold CV	[11]
S5	XGBoost	10-fold CV	[12]
S6	LightGBM, XGBoost	10-fold CV	[13]
S7	RF, Neural networks, LR, XGBoost	CV, AUC, grid search	[14]
S8	RF, gradient boosting machine, SVM	5-fold CV, ROC	[15]
S9	Radiomics-signature model	No formal validation performed	[16]
S10	Multilayer perceptron, SVM, XGBoost	10-fold CV	[17]
S11	Max-p-regions, RF, Jenks natural breaks	RF VIMP^k^ ranking	[18]
S12	CART, RF	Bootstrap sampling	[19]
S13	DT, RF, Boruta feature selection	Confusion matrix	[20]
S14	Bayesian additive, regression trees	Partial dependence plots, variable inclusion proportion	[21]
S15	LR, ridge classifier, SGD^l^classifier, KNN^m^, DT, linear support vector classifier, support vector classifier with radial basis function kernel, Gaussian Naïve Bayes, AdaBoost classifier, RF, gradient boosting, QDA^n^	5-fold CV, LOOCV^o^	[22]
S16	Semiautomated segmentation + conditional LR	80/20 hold-out CV, ROC-AUC, Youden Index	[23]
S17	Random survival forest, Cox proportional hazards	Grid search, C-index^p^	[24]
S18	RF, SVM, gradient boosting machine	10-fold CV	[25]
S19	SVM, DT, naive Bayesian model, and KNN	10-fold CV	[26]

a Lasso: least absolute shrinkage and selection operator.

b LR: logistic regression.

c DT: decision tree.

d SVM: support vector machine.

e CV: cross-validation.

f ROC-AUC: receiver operating characteristic area under the curve.

g XGBoost: extreme gradient boosting.

h LightGBM: light gradient boosting machine.

i CatBoost: categorical boosting.

j CART: classification and regression tree.

k VIMP: variable importance.

l SGD: stochastic gradient descent.

m KNN: *K*-nearest neighbors.

n QDA: quadratic discriminant analysis.

o LOOCV: leave-one-out cross-validation.

p C-index: concordance index.

This review identified a wide array of ML algorithms applied to the analysis of sociodemographic and clinical data related to cancer. Each method presents distinct advantages and limitations, influencing its suitability depending on the specific research context and analytical goals. The most relevant algorithmic approaches are summarized below.

Tree-based methods, particularly RF, were the most frequently used, appearing in 13 of the included studies. RF is widely valued for its interpretability, robustness, and ability to process both categorical and continuous variables, making it especially well-suited to heterogeneous datasets.

Boosting techniques, such as XGBoost and light gradient boosting machine (LightGBM), featured prominently in studies aiming for high predictive accuracy. XGBoost, used in 7 studies, is noted for its computational efficiency and its capacity to manage imbalanced data, while LightGBM is often selected in contexts where large-scale data processing is prioritized.

A smaller subset of studies used Bayesian additive regression trees, which were particularly useful in modeling uncertainty and capturing complex non-linear associations. These features make Bayesian additive regression trees well-suited for analyzing disparities across ethnic and clinical subgroups.

Support vector machines (SVM) appeared in 5 studies and are recognized for their ability to handle high-dimensional data and to separate complex classes using nonlinear decision boundaries [27]. However, their performance is highly dependent on careful hyperparameter tuning, which can be challenging in the presence of large or noisy datasets [27]. Overall, SVM models remain a valuable choice for complex biomedical data when appropriately optimized and validated within diverse clinical contexts.

Artificial neural networks (ANNs) were applied in select studies and demonstrated strong performance in modeling nonlinear relationships and uncovering hidden patterns in complex datasets [28]. Despite their flexibility, the limited interpretability of ANNs often restricts their use in clinical contexts where transparency and explainability are required [28]. Their use, therefore, should be accompanied by complementary interpretability frameworks to ensure clinical reliability and trustworthiness.

Regression-based models, including the least absolute shrinkage and selection operator and ridge regression, were commonly used as baseline models or for feature selection. These methods are appreciated for their simplicity and interpretability, although they may underperform in settings involving nonlinear relationships or intricate interactions between variables [29]. Nevertheless, their transparency and ease of implementation make them a critical reference point for benchmarking more advanced ML models in oncology research.

Some studies also implemented bagged classification and regression tree models and ensemble methods such as stacking, reflecting a methodological interest in combining simplicity with predictive robustness. These strategies reduce model variance and enhance accuracy by integrating multiple base learners.

Overall, the analysis reveals a strong preference for tree-based algorithms, which offer an optimal balance between accuracy, interpretability, and adaptability to real-world clinical data. However, the choice of algorithm varied according to the nature of the dataset and the specific research objectives. More recent studies have increasingly adopted advanced methods such as boosting and neural networks, which provide enhanced predictive power but require greater expertise for interpretation and implementation.

### Common Validation Methods

The reviewed studies showed a strong preference for cross-validation (CV) as the primary strategy to evaluate ML models applied to the identification of sociodemographic factors related to cancer. This approach is widely recognized for its ability to reduce overfitting and enhance the robustness of predictive performance. Several configurations of CV were used across studies, with 10-fold CV being the most commonly used. This method appeared in studies such as Dianati-Nasab et al [24], Stabellini et al [20], and Afrash et al [22], where it facilitated efficient partitioning of data into training and testing subsets, maximizing the use of available datasets.

In some cases, CV was complemented with repeated sampling to mitigate random variation and reinforce consistency. For instance, Wang et al [30] implemented repetitions alongside 10-fold CV to strengthen model reliability. A less frequently used configuration, 5-fold CV, was applied in studies like Kaushik et al [11], offering a computationally efficient alternative without substantially compromising model evaluation.

Several studies further enhanced reliability by incorporating multiple repetitions. A notable example is the work of He et al [9], who used 200 repetitions and evaluated model performance using metrics such as the concordance index and variable importance measures to ensure consistency and interpretability.

The choice of evaluation metrics reflected a balanced interest in both model discrimination and interpretability. The area under the receiver operating characteristic curve was one of the most frequently reported metrics, particularly valued for its ability to quantify discrimination capacity. It was prominently featured in studies such as Dehdar et al [19] and Niell et al [12]. Additionally, accuracy, sensitivity, and specificity were widely reported, especially in studies such as Galadima et al [25] and Lilhore et al [14], as they provided a detailed picture of false positive and false negative rates.

Some researchers adopted tailored interpretability metrics to better understand model behavior. For example, Niu et al [15] used variable inclusion proportions and partial dependence plots to explore the relative importance and marginal effect of predictors, offering deeper insights into model mechanisms. Model optimization also played a critical role in the validation process. Techniques such as grid search were frequently used to fine-tune hyperparameters, as observed in the work of Dehdar et al [19]. In more specialized contexts, such as radiomics applications, validation using pretrained models was implemented, for example, in Dercle et al [21], focusing on metastatic colorectal cancer and highlighting the relevance of domain-specific strategies.

While most studies ensured strong internal validity, a common limitation was the lack of external validation. Although a few studies used unseen datasets or pretrained models to assess generalizability, the overall scarcity of external validation in heterogeneous populations restricts the broader applicability of findings. This underscores the importance of expanding validation practices to include more diverse datasets and real-world scenarios.

### Analysis of Sociodemographic Variables

The reviewed studies demonstrate considerable variability in the types of sociodemographic variables incorporated into oncology research using ML techniques. Individual-level factors, such as age and male or female or intersex, were the most frequently included, underscoring their foundational role in the development and prognosis of various cancer types. For example, in breast cancer research, variables such as age at diagnosis and hormonal status appear consistently, as noted in the studies by Dianati-Nasab et al [24] and Niell et al [12]. Similarly, race and ethnicity were widely explored in studies addressing lung and colorectal cancer [9], highlighting disparities in health outcomes associated with these variables.

In addition to individual characteristics, several studies incorporated socioeconomic and access-related factors, which reflect broader SDH. Educational attainment and household income, often used as proxies for access to health resources and health-seeking behavior, featured prominently in studies on colorectal cancer [13] and advanced-stage breast cancer [13]. Other key access variables, such as transportation availability and type of health insurance, were also frequently considered to assess barriers to diagnosis and treatment, as shown in the works of Wang et al [30] and Afrash et al [22].

Some studies expanded their scope to include community- and environment-level variables, though these remain underrepresented overall. Galadima et al [25], for instance, investigated aspects of the built environment, such as crime rates and housing values, and their association with late-stage colorectal cancer diagnoses. Similarly, Dehdar et al [19] examined the influence of residence location, urban versus rural, on access to medical services, illustrating geographic disparities in health care delivery.

Regarding cancer types, breast cancer was the most frequently studied, followed by colorectal, lung, and gastric cancer. Research on breast cancer often focuses on the impact of delayed diagnosis and racial disparities, as seen in studies by Stabellini et al [20]. In contrast, studies on colorectal cancer emphasized socioeconomic factors and health care access, particularly in relation to late-stage detection [13,25]. Lung cancer studies primarily explored racial disparities and quality-of-life indicators in survival prediction [9,10].

**Figure 2.**
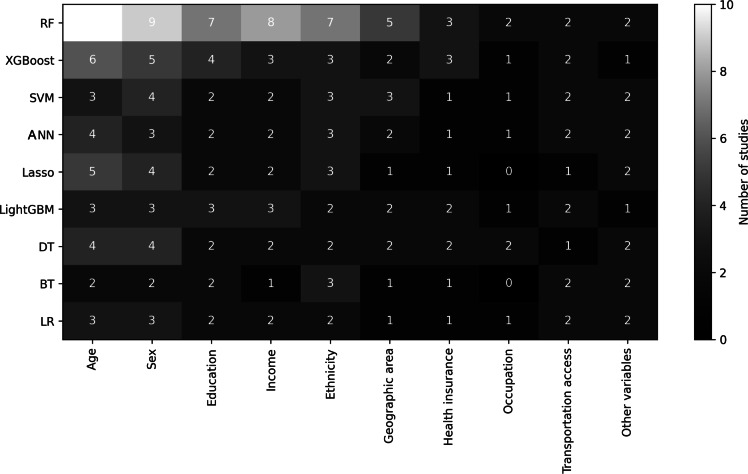
Association between machine learning techniques and sociodemographic variables. ANN: artificial neural network; BT: Bayesian tree; DT: decision tree; LASSO: least absolute shrinkage and selection operator; LightGBM: light gradient boosting machine; LR: logistic regression; RF: random forest; SVM: support vector machine; XGBoost: extreme gradient boosting.

A few studies adopted a broader, multicancer approach, examining sociodemographic patterns across different tumor types. For example, Stabellini et al [17] analyzed unplanned hospital readmissions in patients with solid tumors, integrating sociodemographic variables that have a direct influence on health outcomes. To provide a visual synthesis of these findings, Figure 2 presents a summary linking the ML algorithms used with the most frequently analyzed sociodemographic variables.

## Discussion

### Stratification of Findings

The reviewed studies confirm the potential of ML to identify patterns of predictive relevance of sociodemographic variables in relation to oncologic outcomes. However, the evidence remains fragmented and heterogeneous, with limited integration of contextual factors, reliance on predominantly internal validation, and little standardization in the reporting of performance and fairness. Overall, the findings suggest that ML can enhance risk stratification and the detection of disparities, but its real impact depends on methodological decisions that currently remain inconsistent.

In breast cancer, models most often prioritize age, race or ethnicity, and socioeconomic proxies to explain adverse events and late diagnosis. In colorectal cancer, income, insurance coverage, and geographic location are central for predicting advanced stage and survival. In lung cancer, studies more frequently explore ethnic disparities and quality-of-life measures associated with prognosis. This diversity suggests that the relevant set of SDoH is tumor-specific and linked to each care pathway.

Retrospective studies dominate; while they provide volume and feasibility, they limit causal inference and the ability to adapt to temporal social changes (eg, economic shocks, migration, or health system reforms). Prospective and longitudinal cohort designs would better capture the temporal variability of SDoH.

Greater interpretative weight should be placed on studies with stronger control of confounding, explicit handling of missing data, subgroup analyses, and when available, external validation. In contrast, studies with incomplete reporting of variables and opaque pipelines should be viewed as exploratory signals rather than evidence ready for implementation.

### Linking Inequities and ML Limitations

When sociodemographic factors are omitted or inconsistently defined, ML models often end up reflecting pre-existing inequities in access to and quality of care instead of uncovering or addressing them. This reflection of structural disparities undermines both the external validity and the generalizability of predictive models [31,32]. Evidence from recent reviews indicates that algorithmic bias in health care typically emerges from unbalanced data representation and the absence of systematic fairness assessments, highlighting the importance of transparency and interpretability in model design [33,34]. Although variable-importance analyses can reveal which sociodemographic features most influence predictions, they fall short of explaining underlying causal mechanisms. As Prosperi et al [35] and McCradden et al [36] emphasize, achieving fairness and accountability in ML-driven health applications requires methodological and ethical frameworks that move beyond conventional supervised learning. For this reason, throughout this review, the term “associated factors” is used exclusively in a predictive, not causal, sense.

To advance the field, it is essential to standardize the reporting of sociodemographic variables including age, male or female or intersex, race or ethnicity, education, income, rurality, and health insurance as a minimum dataset to reduce heterogeneity and enable comparability across studies. Fairness metrics, such as demographic parity, equal opportunity, and subgroup calibration, should be applied alongside conventional measures like area under the curve and accuracy to explicitly assess model performance in vulnerable populations. Routine multicenter external validation is needed, testing models across diverse geographical and socioeconomic contexts. Incorporating neighborhood-level data (eg, area-level socioeconomic indices, transportation access, and housing conditions) can provide valuable context for individual predictors. Interdisciplinary collaboration between data scientists, oncologists, public health practitioners, and experts in social science and policy should be promoted to ensure that models achieve both technical precision and equity. Finally, transparent dissemination, including open-source code and model cards documenting limitations, is crucial to strengthen reproducibility and accountability.

### Principal Findings

This systematic review synthesized evidence from 19 primary studies published between 2018 and 2024 that applied ML techniques to analyze sociodemographic factors associated with cancer. The analysis revealed consistent methodological patterns, frequently used variables, and prevalent validation strategies, while also identifying key implications for both academic research and professional practice.

From a methodological perspective, there was a strong preference for tree-based algorithms, particularly RF, which was the most frequently used due to its capacity to manage heterogeneous datasets while preserving a degree of interpretability. Boosting methods, notably XGBoost and LightGBM, were also prominent, especially in studies aiming for high predictive accuracy in high-dimensional or imbalanced data contexts. Less frequently, SVMs and ANNs were used to capture complex, nonlinear relationships, typically in specialized modeling scenarios. Regression-based approaches such as the least absolute shrinkage and selection operator and Ridge regression were primarily used for feature selection or as baseline models for comparative purposes.

Across the studies, a consistent set of core sociodemographic variables was identified. The most commonly included were age, male or female or intersex, educational level, income, ethnicity, and geographic location. These factors were primarily used to predict diagnostic timelines, disparities in access to treatment, and survival outcomes. However, only a limited number of studies incorporated broader structural or contextual variables—such as neighborhood characteristics, transportation access, or housing conditions—that could enrich model performance by capturing deeper dimensions of health inequity.

In terms of validation strategies, 10-fold CV was the most frequently implemented, followed by 5-fold validation in settings with limited computational resources. Most studies relied on standard evaluation metrics such as accuracy, area under the receiver operating characteristic curve, and sensitivity or specificity, reflecting a predominant focus on internal performance. However, the use of external validation with independent datasets was rare, limiting the generalizability of findings to broader, more diverse populations and real-world clinical environments.

From an applied perspective, the findings suggest that ML holds significant promise for identifying and quantifying structural health disparities in oncology. For the academic research community, this review highlights the importance of developing models that explicitly integrate SDoH, moving beyond individual-level data to encompass contextual and systemic influences. For clinicians and policymakers, predictive models incorporating sociodemographic factors offer a valuable complement to traditional clinical assessments, enabling the early identification of at-risk populations who might otherwise be overlooked.

Taken together, these findings underscore the transformative potential of ML when applied with methodological rigor, interpretability, and an explicit commitment to equity. Advancing this field will require not only continued technical innovation, but also interdisciplinary collaboration and a deliberate focus on addressing the social and structural dimensions of cancer prevention, diagnosis, and care.

### Limitations

We critically assessed potential threats to the validity of our SLR based on the Wohlin classification, which provides clear guidelines for identifying and mitigating such threats [37].

Internal validity threats involve factors that could influence the reliability and accuracy of our study outcomes. A primary concern is selection bias, potentially stemming from limitations inherent in our search strategy and inclusion criteria. To minimize this risk, we carefully defined explicit and rigorous inclusion and exclusion criteria, conducting systematic searches across multiple reputable academic databases. Despite these measures, the relatively small final sample size (N=19) remains a limitation. To further reinforce internal validity, we conducted independent cross-checking and reviews with three domain experts, ensuring consistency and reliability in the selection and evaluation of studies.

External validity threats refer to the generalizability of our findings beyond the specific studies reviewed. A significant concern here is the representativeness of the primary studies regarding the broader application of ML to sociodemographic determinants of cancer. To mitigate this threat, we engaged external experts in data science and public health to provide critical insights and feedback on our findings, enhancing the relevance and applicability across different contexts [7].

Finally, construct validity threats pertain to the accurate interpretation and generalization of results in alignment with the study objectives. The primary concern here is potential subjectivity or bias in interpreting the findings. To address this, external collaborators participated in the analysis and classification phases, providing independent perspectives that strengthened the robustness and objectivity of our conclusions.

### Comparison With Prior Work

Several systematic reviews have examined the application of ML techniques in oncology, but their scope differs significantly from this study. Adeoye et al [38] evaluated ML models in oncology settings with limited resources, identifying gaps in external validation and clinical adoption, but without providing a detailed analysis of sociodemographic variables. Hossain Raju et al [26] reviewed the use of deep learning for breast cancer risk prediction, focusing mainly on imaging and genomic data. Kumar et al [39] offered a broad overview of AI in oncology, emphasizing technical innovation rather than social determinants. Zeinali et al [40] analyzed the application of ML in predicting cancer-related symptoms, again with a focus on clinical variables.

In addition, recent editorials and reviews have highlighted the need to move toward more interpretable and explainable models. For example, Hrinivich et al [4] warned about the risks associated with the lack of interpretability in ML models in oncology, noting that reliance on opaque systems may amplify biases and weaken clinical trust. However, while these works underscore the importance of technical transparency, they do not systematically address the incorporation of sociodemographic factors into predictive cancer models.

Our review differs from previous contributions in three main ways. First, we provide a systematic synthesis of primary studies in which sociodemographic factors are explicitly integrated into ML models applied to oncological outcomes, thereby moving beyond an exclusively clinical or technical lens. Second, we critically assess methodological limitations—such as the lack of external validation, limited interpretability, and absence of fairness metrics—specifically in relation to the inclusion of sociodemographic data. Third, we connect these findings to broader discussions of equity and public health, emphasizing that neglecting social determinants may inadvertently reinforce inequalities in cancer care. By placing sociodemographic factors at the center rather than at the periphery, this review addresses an underexplored yet essential dimension of the field.

Ultimately, our findings contribute meaningfully to the growing body of literature by illustrating how ML can be leveraged to deepen our understanding of social inequalities in cancer outcomes. Rather than treating sociodemographic variables as peripheral, this study brings them to the forefront of analysis, offering a more nuanced view of how structural and contextual factors shape cancer risk, access to care, and treatment outcomes. These insights can help guide the development of more inclusive health policies and inform interventions that are responsive to the realities of diverse and historically underserved populations.

### Conclusions

This review indicates that the integration of sociodemographic factors into ML models for oncology is still an emerging field, with a modest evidence base that appears to be steadily growing. Only 19 primary studies met our inclusion criteria, yet their collective findings point to the potential benefits of embedding these variables within predictive frameworks. There is some evidence to suggest that explicitly accounting for sociodemographic factors could refine predictive accuracy and fairness, although these associations remain noncausal. That said, such conclusions remain tentative, as further research is needed to substantiate these observations. Looking ahead, researchers might prioritize enhancing the transparency of these models, exploring fairness metrics, and considering how such tools align with the broader goals of health policy. Advancing these aspects could prove vital in ensuring that ML supports both precision oncology and equitable public health outcomes. It is worth noting that, although the variables examined in this review are those most frequently reported in existing datasets, future research could benefit from incorporating contextual and structural determinants to strengthen both fairness and interpretability in ML-based cancer studies (MultimediaAppendices 2  3).

## Supplementary material

10.2196/79187 Multimedia Appendix 1 Quality assessment criteria and the assignment of scores.

10.2196/79187 Multimedia Appendix 2 Primary studies by year and publication type.

10.2196/79187 Multimedia Appendix 3 Primary studies description.

10.2196/79187 Checklist 1 PRISMA 2020 checklist.

## Abbreviations

AI: artificial intelligence
ANN: artificial neural network
LightGBM: light gradient boosting machine
ML: machine learning
PICO: population, intervention, comparison, and outcome
PRISMA: Preferred Reporting Items for Systematic Reviews and Meta-Analyses
RF: random forest
SDoH: social determinants of health
SLR: systematic literature review
SVM: support vector machine
XGBoost: extreme gradient boosting
