# Supplementary material for: Machine Learning Techniques Used for the Identification of Sociodemographic Factors Associated With Cancer: Systematic Literature Review
Source: J Med Internet Res. 2026 Jan 28;28:e79187. doi: 10.2196/79187 (PMC12851563; doi:10.2196/79187)
Supplement: Multimedia Appendix 1 [file jmir-v28-e79187-s001.docx]

## **Multimedia Appendix 1: Quality assessment criteria and the assignment of scores.**

| ID | Quality assessment criterion | Answer | Points |
| --- | --- | --- | --- |
| QA1 | Clearly states the purpose of the study regarding the use of Machine Learning techniques to identify sociodemographic factors associated with cancer | Satisfied | 2 |
|  |  | Neutral | 1 |
|  |  | Dissatisfied | 0 |
| QA2 | Method Description | Standards, well documented guidelines or software development methodologies. | 2 |
|  |  | Detailed ad-hoc method | 1 |
|  |  | Information not specified | 0 |
| QA3 | Rigor of the Method | Controlled Experiment | 2 |
|  |  | Study case, survey or quality assessment | 1 |
|  |  | Information not specified | 0 |
| QA4 | Adoption or Application | Applied in a real context | 2 |
|  |  | Applied in a simulated context | 1 |
|  |  | Information not specified | 0 |
| QA5 | Quantitative results | Results are explained from quantitative results obtained from the research | 2 |
|  |  | Results are explained from qualitative results obtained from the research | 1 |
|  |  | Information not specified | 0 |
| QA6 | Scope and limitations discussion | Describes threats that could invalidate the work | 2 |
|  |  | Describes scope and restrictions but does not specify threats | 1 |
|  |  | Information not specified | 0 |
