# Supplementary figures and images for: Machine Learning Techniques Used for the Identification of Sociodemographic Factors Associated With Cancer: Systematic Literature Review
Source: J Med Internet Res. 2026 Jan 28;28:e79187. doi: 10.2196/79187 (PMC12851563; doi:10.2196/79187)

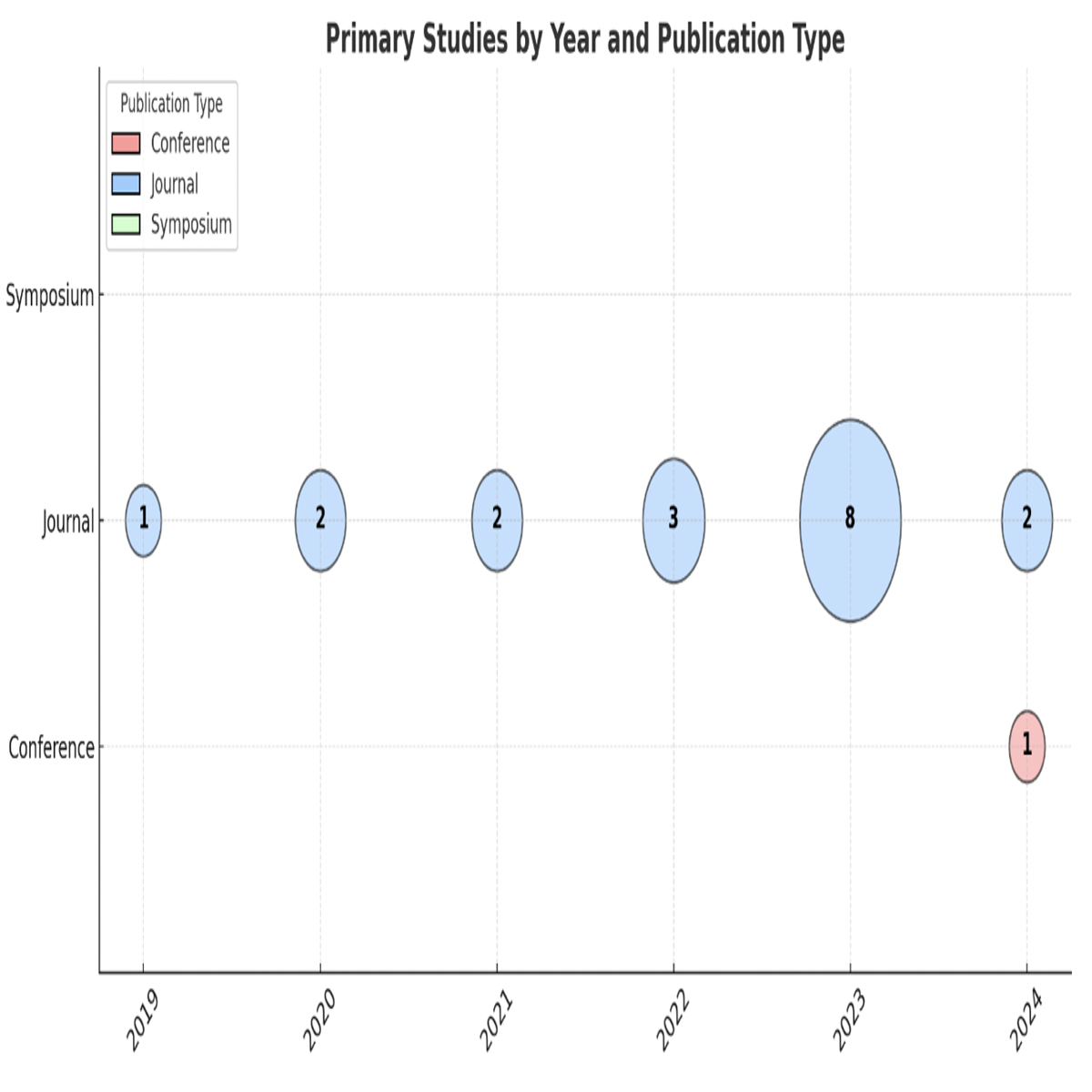

Supplement: Multimedia Appendix 2 [file jmir-v28-e79187-s002.png]
