# Supplementary material for: Machine Learning Techniques Used for the Identification of Sociodemographic Factors Associated With Cancer: Systematic Literature Review
Source: J Med Internet Res. 2026 Jan 28;28:e79187. doi: 10.2196/79187 (PMC12851563; doi:10.2196/79187)
Supplement: Multimedia Appendix 3 [file jmir-v28-e79187-s003.docx]

**Multimedia Appendix 3: Primary studies description**

| ID | Year | Authors | Country | Paper | Venue | Ref. |
| --- | --- | --- | --- | --- | --- | --- |
| S1 | 2019 | Asghar Mortezagholi, Omid Khosravizadeh, Mohammad Bagher Menhaj, Younes Shafigh, Rohollah Kalhor | Iran | Make Intelligent of Gastric Cancer Diagnosis Error in Qazvin's Medical Centers: Using Data Mining Method | Asian Pacific Journal of Cancer Prevention | [23] |
| S2 | 2020 | Jiangping He, James X. Zhang, Chin-tu Chen, Yan Ma, Raymond De Guzman, Jianfeng Meng, Yonglin Pu. | China and the United States | The Relative Importance of Clinical and Socio-demographic Variables in Prognostic Prediction in Non-Small Cell Lung Cancer: A Variable Importance Approach | Medical Care | [16] |
| S3 | 2020 | Jin-ah Sim, Young Ae Kim, Ju Han Kim, Jong Mog Lee, Moon Soo Kim, Young Mog Shim, Jae Ill Zo, Young Ho Yun | South Korea | The major effects of health-related quality of life on 5-year survival prediction among lung cancer survivors: applications of machine learning | Scientific Reports | [25] |
| S4 | 2021 | Manoj Kaushik, Rakesh Chandra Joshi, Atar Singh Kushwah, Maneesh Kumar Gupta, Monisha Banerjee, Radim Burget, Malay Kishore Dutta | India | Cytokine gene variants and socio-demographic characteristics as predictors of cervical cancer: A machine learning approach | Computers in Biology and Medicine | [15] |
| S5 | 2021 | Bethany L. Niell, Mahmoud Abdalah, Olya Stringfield, Natarajan Raghunand, Dana Ataya,  Robert Gillies, Yoganand Balagurunathan | United States | Quantitative Measures of Background Parenchymal Enhancement Predict Breast Cancer Risk | AJR American Journal of Roentgenology | [18] |
| S6 | 2022 | Weichuan Dong, Wyatt P. Bensken, Uriel Kim, Johnie Rose, Nathan A. Berger, Siran M. Koroukian | United States | Phenotype Discovery and Geographic Disparities of Late-Stage Breast Cancer Diagnosis across U.S. Counties: A Machine Learning Approach | Cancer Epidemiology, Biomarkers & Prevention | [24] |
| S7 | 2022 | Mesh Kumar Lilhore, M. Poongodi, Amandeep Kaur, Sarita Simaiya, Abeer D. Algarni, Hela Elmannai, V. Vijayakumar, Godwin Brown Tunze, Mounir Hamdi | India | Hybrid Model for Detection of Cervical Cancer Using CausalAnalysis and Machine Learning Techniques; | Computational and Mathematical Methods in Medicine | [20] |
| S8 | 2022 | Li Niu, Liangyuan Hu, Yan Li, Bian Liu | United States | Correlates of cancer prevalence across census tracts in the United States: A Bayesian machine learning approach | Spatial and Spatio-temporal Epidemiology | [21] |
| S9 | 2023 | Nickolas Stabellini, Mantas Dmukauskas, Marcio S. Bittencourt, Jennifer Cullen, Amie J. Barda, Justin X. Moore, Susan Dent, Husam Abdel-Qadir, Aniket A. Kawatkar, Ambarish Pandey, John Shanahan, Jill S. Barnholtz-Sloan, Kristin A. Waite, Alberto J. Montero, Avirup Guha | United States | Social Determinants of Health and Racial Disparities in Cardiac Events in Breast Cancer | Journal of the National Comprehensive Cancer Network | [33] |
| S10 | 2023 | Nickolas Stabellini, Aziz Nazha, Nikita Agrawal, Merilys Huhn, John Shanahan, Nelson Hamerschlak, Kristin Waite, Jill S. Barnholtz-Sloan, Alberto J. Montero | United States | Thirty-Day Unplanned Hospital Readmissions in Patients With Cancer and the Impact of Social Determinants of Health: A Machine Learning Approach | JCO Clinical Cancer Informatics | [26] |
| S11 | 2023 | A. Stone, C. Kalahiki, L. Li, N. Hubig, F. Iuricich, H. Dunn | United States | Evaluation of breast tumor morphologies from African American and Caucasian patients | Computational and Structural Biotechnology Journal | [32] |
| S12 | 2023 | Samira Dehdar, Khodakaram Salimifard, Reza Mohammadi, Maryam Marzban, Sara Saadatmand, Mohammad Fararouei, Mostafa Dianati-Nasab | Iran | Applications of different machine learning approaches in prediction of breast cancer diagnosis delay | Frontiers in Oncology | [17] |
| S13 | 2023 | Nickolas Stabellini, Jennifer Cullen, Justin X. Moore, Susan Dent, Arnethea L. Sutton, John Shanahan, Alberto J. Montero, Avirup Guha | United States | Social Determinants of Health Data Improve the Prediction of Cardiac Outcomes in Females with Breast Cancer | Cancers | [12] |
| S14 | 2023 | Laurent Dercle, Melissa Yang, Mithat Gönen, Jessica Flynn, Chaya S. Moskowitz, Dana E. Connors, Hao Yang, Lin Lu, Diane Reidy-Lagunes, Tito Fojo, Sanja Karovic, Binsheng Zhao, Lawrence H. Schwartz, Brian S. Henick. | United States | Ethnic diversity in treatment response for colorectal cancer: proof of concept for radiomics-driven enrichment trials | European Radiology | [22] |
| S15 | 2023 | Mohammad Reza Afrash, Mohsen Shafiee, Hadi Kazemi-Arpanahi | Iran | Establishing machine learning models to predict the early risk of gastric cancer based on lifestyle factors | BMC Gastroenterology | [13] |
| S16 | 2023 | Weichuan Dong, Uriel Kim, Johnie Rose, Richard S. Hoehn, Matthew Kucmanic, Kirsten Eom, Shu Li, Nathan A. Berger, Siran M. Koroukian | United States | Geographic Variation and Risk Factor Association of Early Versus Late Onset Colorectal Cancer | Cancers | [34] |
| S17 | 2024 | Mostafa Dianati-Nasab, Khodakaram Salimifard, Reza Mohammadi, Sara Saadatmand, Mohammad Fararouei, Kosar S. Hosseini, Behshid Jiavid-Sharifi, Thierry Chaussalet, Samira Dehda | Iran | Machine learning algorithms to uncover risk factors of breast cancer: insights from a large case-control study | Frontiers in Oncology | [11] |
| S18 | 2024 | Hadiza Galadima, Rexford Anson-Dwamena, Ashley Johnson, Ghalib Bello, Georges Adunlin y James Blando | United States | Machine Learning as a Tool for Early Detection: A Focus on Late-Stage Colorectal Cancer across Socioeconomic Spectrums | Cancers | [19] |
| S19 | 2024 | Azad Hossain Raju, Jahirul Islam, Touhid Imam, Mohammad Navid Nayyem, Abdullah Al Rakin, Mohammad Shihab Uddin | United States | An Ontological Framework for Lung Carcinoma Prognostication via Sophisticated Stacking and Synthetic Minority Oversampling Techniques | IEEE Conference | [29] |
